# The case for mandatory reporting to enable targets for healthy and environmentally sustainable food: means, motive and opportunity

**DOI:** 10.1186/s12916-026-04690-6

**Published:** 2026-02-10

**Authors:** Rachel Pechey, Lauren Bandy, Susan A. Jebb

**Affiliations:** Nuffield Department of Primary Care Health Sciences, Radcliffe Observatory Quarter, University of Oxford, Oxford, OX2 6GG UK

**Keywords:** Mandatory reporting, Health, Targets, Food

## Abstract

**Background:**

Evidence suggests we must change both the type of food we consume and the way we produce it, at a transformational scale, to protect population and planetary health and avoid exacerbating existing diet-related health inequalities.

**Discussion:**

We summarise key findings from behaviour change theory and literature, highlighting the need for means, motive and opportunity to enact behaviour change. We evaluate and contrast the implications for interventions aimed at individuals vs. businesses, arguing that policy must shift focus from individual responsibility to systemic change.

**Conclusion:**

Past public health interventions have tended to focus on individuals’ motivation, with limited impact, while interventions that target the motivation of businesses, if enacted, would likely garner substantially greater impact. Governments implementing mandatory reporting could provide the foundation to realign the incentives that shape business practices. This would subsequently enable mandatory targets to be introduced on foods sold, providing in turn the necessary conditions — the means and opportunity — for individuals to enact dietary change.

## Background

Poor diet is a major risk factor for disease and premature death in the UK and globally [1] and is not evenly distributed across the population [2]. Meanwhile, greenhouse gas emissions from the food system already make up one third of all global emissions and are increasing [3]. It is now well accepted that we must change both the type of food we consume and the way we produce it, at a transformational scale, to protect population and planetary health and avoid exacerbating existing diet-related health inequalities [4].

## Main text

### Means, motive and opportunity

There have been significant efforts to encourage healthier and more environmentally sustainable eating habits [5]. Yet, much of the research has focused heavily on educating people or providing information to drive dietary change [5]. These efforts have only led to, at best, modest improvements to population diet [6].

The COM-B model — a behavioural science framework — emphasises that changing behaviour requires people to have not only the motivation, but also the capability and opportunity to enact this behaviour (means, motive and opportunity) [7]. This concept is echoed in socioecological models, which view individuals as sitting at the centre of a nested set of increasingly broader influences, including social environments, physical environments and wider cultural norms and regulatory systems [8]. As such, interventions that aim solely to boost people’s motivation to behave differently (e.g. to purchase healthier food) are unlikely to succeed if systemic barriers persist that impact their means (e.g. relatively higher costs of healthier food) or opportunity to enact these (e.g. limited availability in local stores).

**Fig. 1:**
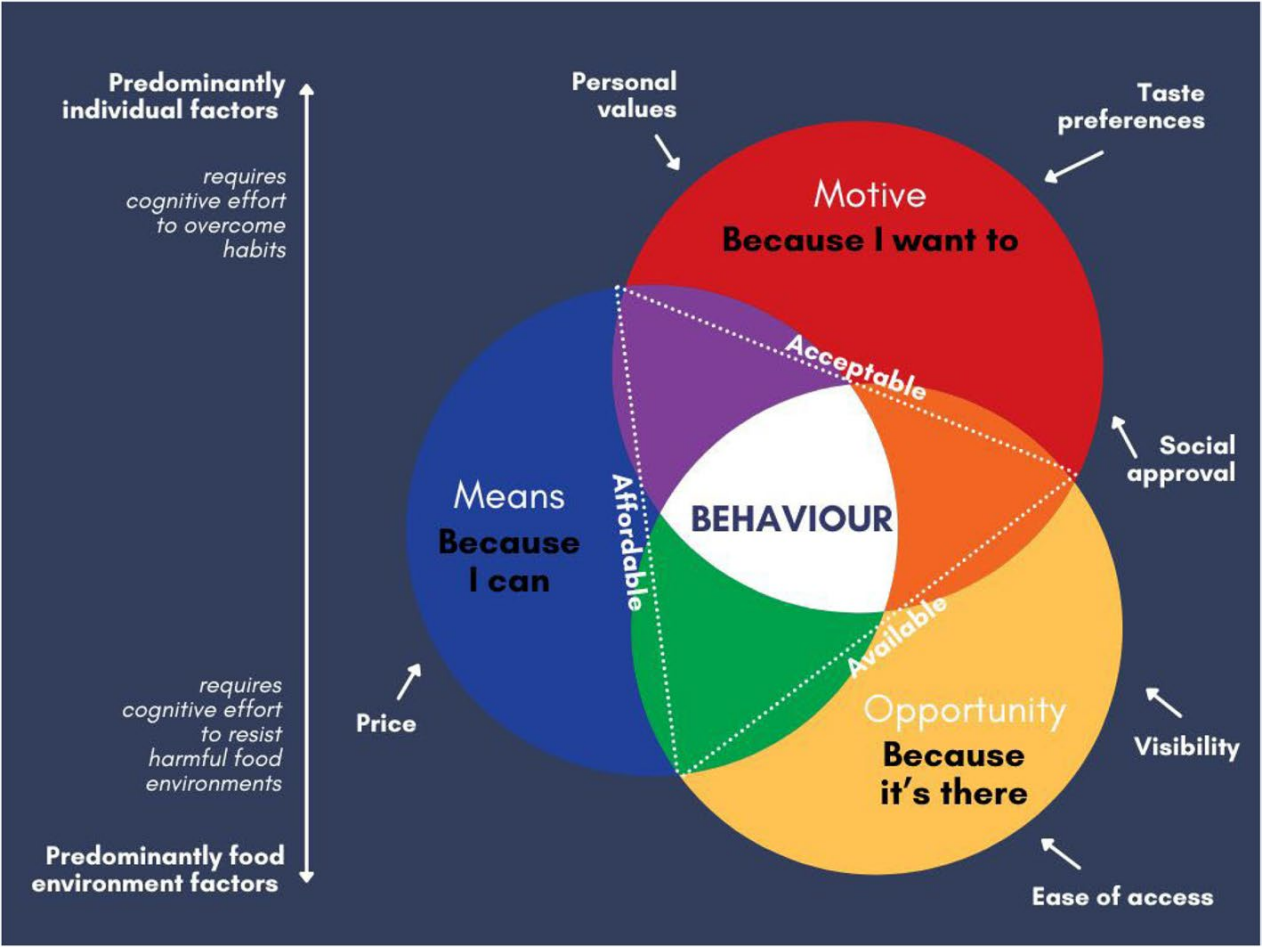
Means, motive and opportunity in the context of dietary behaviour within a food environment that promotes less healthy and less environmentally sustainable options. People will choose a food only if it is affordable, acceptable and available to them (passing a personal minimum threshold (dotted white lines)). All three factors (sufficient means, motive and opportunity — i.e. being affordable, acceptable and available) need to be met. However, the degree to which each of the three factors is determined by predominantly individual vs. predominantly environmental factors varies. Examples of some key factors influencing means, motive and opportunity within food contexts are shown*.* Figure created using canva.com

Changing food environments, such as within shops, restaurants and schools, can be effective [9]. Governments have the means to directly mandate the options that can be offered in public settings (e.g. healthy food standards in schools). However, sales by retailers account for the majority of food purchases — demonstrating a clear opportunity to achieve substantive changes — but policies targeting the private sector are harder for governments to enact. There is a need for policies to boost access to more beneficial foods and/or limit access to options associated with potential harms (see Fig. 1).

These policies may also be more equitable than simply providing information [10]: Changing the food environment can influence the behaviour of everyone who interacts with it, regardless of their personal motivations. In contrast, providing information to individuals (e.g. via labels on products in a supermarket) has an opportunity cost for each individual, considering the time and mental energy required to engage in a way that is meaningful and sustained. Limited time or financial resources, together with personal stressors, act as barriers to engagement and are not equitably distributed across the population [11].

Until now there has been limited policy action targeting food environments — with some exceptions, such as restrictions on the promotions of less healthy foods in the UK [12]. The onus instead has largely been placed on individuals to change. Yet while consumers can be powerful levers of change, as businesses seek to gain financially from attracting their support [13], positive momentum is thwarted if the system does not provide the available, affordable and acceptable healthy and environmentally sustainable food that would enable customers to act. This is reflected by calls within behavioural science to move towards interventions targeting systems rather than individuals [14].

### Targeting business

While motivating individuals to change behaviour in the absence of food system change has been largely unsuccessful, motivating individuals who are acting on behalf of businesses is different. Compared to habitual consumer selections, business decisions, particularly within large enterprises, are likely to be strategic and deliberative, taking careful account of their operating context. Public health policy needs to target the motivations of these key food system players, who hold important levers to unlocking population behaviour change.

Governments have the option of mandating changes to industry behaviour via regulations to alter specific aspects of food environments. These could include restrictions on the promotion of less healthy foods (for example, limiting their display in prominent areas, such as aisle ends in supermarkets; implemented in the UK), bans on advertising of less healthy products at certain times and in particular settings (as in Chile), or ensuring the availability of healthier or more sustainable options, such as requiring vegan food options in restaurants (as enacted in Portugal). Such moves would clearly be useful steps to incrementally alter food environments.

However, no one policy by itself has achieved the necessary level of change. A possible alternative or complement to this approach is to set targets for retailers and other key industry players, with the aim of shifting their nutritional and environmental performance. This — like other food policies — is likely to be subject to pushback or lobbying from industry, a key barrier to change [4]. However, once introduced, it could shift corporate focus onto the key public health outcomes of interest. Better aligning corporate interests with public health goals could lessen the use of loopholes — such as some food businesses replacing HFSS foods with alcohol when UK prominent placement restrictions came into force [15] — or the need for continued updating of legislation, e.g. in response to new digital marketing practices. The challenge of overcoming industry pressure will require strong leadership and a careful programme of work to decide what precisely should be measured, how targets are set up and how they will be enforced.

### Step 1: Mandatory reporting

The first step is to require companies to disclose their impact in the food system, through mandatory reporting [16]. The first country to announce plans for implementation with regard to healthiness of food sales — England — did so in July 2025 [17]. This will allow for benchmarking across companies, based on the healthiness of their product portfolios and business practices, providing transparency for civil society [18], allowing investors to assess the level of risk companies are exposed to in terms of potential legislation [19], and giving policymakers an overview of how companies compare and with which to assess progress.

Details as yet are scant as to how this would be implemented in England, and there needs to be a careful approach to ensure potential benefits are realised. Appropriate metrics need to be agreed, maintaining consultation with industry, but without watering down of ambition, as has happened with previous initiatives [20]. Metrics need to: complement existing health metrics; ensure a focus on less healthy foods (i.e. in addition to healthier foods); be applicable to all food industry players — retailers, manufacturers and the out-of-home sector — and minimise the burden for businesses (by focusing on data they already need to hold). As an example, these could include the proportion of total sales by volume from less healthy products, as defined by existing nutrient profiling models. Sales of ruminant meat by volume could serve as a proxy indicator until a comprehensive model is available to assess environmental impact. Reporting should also include indicators relating to how well companies are serving the most deprived areas, allowing the potential impact on health inequalities to be monitored. A roadmap of how to progress from current data availability to consistent reporting of the needed outcomes will need to be set out for each of the different sectors (given the richness of data already collected by retailers, in stark contrast to some out-of-home sector businesses). Existing benchmarking initiatives, e.g. by ATNi or NGO Questionmark, could potentially act as complementary resources or there could be independent verification [19].

### Step 2: Target setting

Mandatory reporting allows for the logical next step to drive further change: setting targets. Business practice relies heavily on targets and key performance indicators (KPIs), and governments have previously tried to harness this strategy to encourage changes in behaviour. However, the process will need to be refined. Setting targets requires careful thought to the scale of ambition and to ensure they can be measured and enforced.

Data will be key: An accurate dataset, based on mandatory reporting of specific metrics, enables targets to be precisely defined, taking account of differing baselines and practices within food system players (e.g. manufacturers, retailers, food service providers). Mandatory reporting also provides the basis for monitoring progress towards targets and potentially for any enforcement action. The use of fiscal incentives or penalties, dependent on adherence within a specified timescale, are not without precedent in other areas of the food system and show promise in driving change — for example the successful soft drink industry levy (SDIL) and recent alcohol duty rate threshold changes, designed to encourage product reformulation [21, 22]. If targets are to be enforced there needs to be a clear process to assure the data quality and to ensure a level playing field across industry, given industry tend to self-report higher levels of adherence than independent observations [23].

Previous voluntary targets have not been sufficient to produce change. For instance, the UK’s voluntary salt reduction targets, introduced in 2006, initially led to a drop in salt consumption across multiple categories, but progress later stalled — or even reversed — when the frequency of reporting on outcomes diminished [24]. Targets for sugar and calories, based on a standard 20% cut, achieved only modest improvements in some specific categories because, for many categories, such large changes were unfeasible [25, 26], highlighting the need for careful target setting and tailoring. It is critical that targets are demonstrably realistic and also enforceable to command attention from businesses.

There are roles for researchers to provide evidence to support promising approaches, enable independent monitoring and maintain awareness of lobbying activities. Researchers could help independently evaluate interventions industry puts in place to work towards targets, and to study the effects of combinations of interventions. Developing independent means and measures for validating reported figures, and strengthening publicly available data to allow monitoring changes over time, will be crucial to ensure robust evaluation and enforcement.

### Potential for benefit

Setting targets and monitoring progress can recognise the contribution of the more progressive companies, while penalising the laggards; a point evident in the increasing calls from some businesses for government action [27]. Businesses may prefer and are likely to be better placed than government to know how to manage their portfolios of products towards better nutritional and environmental outcomes. They have the infrastructure, skills and knowledge (i.e. means and opportunity) to make healthy and environmentally sustainable food items that are appealing to their customers. It could potentially help ease the regulatory burden created by a series of policies intended to create a healthier or more environmentally sustainable food environment and sustain high levels of competition in the system that benefit consumers.

Careful implementation of the new proposals for mandatory reporting and targets could provide a supportive regulatory environment with the potential to align motivations and shift the balance of decision making to socially responsible outcomes (corporate responsibility vs. profitability of unhealthy food products), which has not happened organically, even when individual business leaders profess support. These active solutions could help free the food system from the ‘junk food cycle’ where it is currently ensnared, and create a new business model where greater investment in healthier options drives further consumer demand.

### Public support

The need to make this switch to focus on food system players goes beyond just considering the effectiveness of the interventions themselves. Policy implementation is facilitated by public support [28, 29], and government policies targeting retailers and other key industry players are likely to garner greater public support by targeting business practices rather than consumers [30]. This may help overcome potentially problematic higher public support for less-effective interventions that target information or education compared to those targeting food environments [30, 31], where the presence of a less costly (in terms of effort or financial resources) option may lessen support for a costlier but potentially more effective option [14, 32]. Switching focus away from consumers to target food system players could bring gains in public support.

In addition, new policies can lead to a change in attitudes, as happened in the UK when public support for smoking restrictions increased *following* the ban on smoking in public places [33]. As food environments change — with fewer adverts, reduced in-store visibility and less availability of less healthy or less environmentally sustainable options — there is a plausible hypothesis (and preliminary evidence) that perceptions of the popularity of these foods will decline [34] which over time could alter social norms and create a positive feedback loop.

## Conclusions

The current food system drives widespread diet-related ill health, environmental damage and deepening social and economic inequalities. Transformative action is urgently needed to redesign our food environments that currently normalise and encourage overconsumption, particularly of products that undermine public health. Policy must shift focus from individual responsibility to systemic change. We argue that governments implementing mandatory reporting could provide the foundation to realign the incentives that shape business practices. By changing the motive of businesses through incentivising the production and sale of affordable, nutritious and environmentally sustainable foods, policies can create the necessary conditions — namely, means, motive and opportunity — for consumers to act, and thereby maintain healthier and more environmentally sustainable diets.

## Data Availability

Not applicable
